# Severe Air Pollution Exposure and Long-Term Health Outcomes

**DOI:** 10.3390/ijerph192114019

**Published:** 2022-10-27

**Authors:** Younoh Kim, Vlad Radoias

**Affiliations:** Department of Economics and International Business, Sam Houston State University, 1905 University Ave, Huntsville, TX 77340, USA

**Keywords:** air pollution, health, lung capacity, general health status

## Abstract

Background: There is a large literature that documents the negative health implications of exposure to air pollution, particularly PM2.5. Much of this literature, however, relies on short-term cross-sectional data, which cannot establish a true causal link between pollution and health. There are also very few studies that document long- and very long-term effects. Purpose: This study intends to estimate a causal relationship between exposure to severe air pollution and negative health outcomes that persist over long periods of time. Methods: We use a large longitudinal dataset that spans almost 2 decades and that allows us to not only document the persistence of negative health effects, but also a pattern of recovery from a severe pollution episode. We use multivariate regression methods to estimate a causal link between air pollution and health over time. A large pollution shock that occurred in 1997 in Indonesia is used as a natural experiment to pinpoint the true causal effects of pollution exposure and not mere correlations. Results: Exposure to an additional unit of pollution in 1997 leads to a loss of roughly six units of lung capacity and to an increase of 4.3% in the probability of being in poor general health, as measured ten years after the pollution exposure. These effects somewhat diminish over time, to a loss of roughly three units of lung capacity and to an increase of only about 3% in the probability of being in poor general health, as measured 17 years after exposure. Conclusions: Our study finds significant health consequences of exposure to air pollution, which persist over long periods of time, with some patterns of recovery. Policymakers should pay special attention to such massive sources of pollution and try to mitigate these negative health consequences.

## 1. Introduction

A vast strand of literature [1,2,3] shows that air pollution in the form of fine particulate matter (PM 2.5) is strongly linked to a series of negative health outcomes, especially when children or the elderly are concerned. Air pollution contributes to the deaths of millions of people per year, including hundreds of thousands of children [4]. Exposure to air pollution is associated with respiratory infections [5], deficits in lung function [6], asthma [7], COPD, and even respiratory cancer [8]. Air pollution also leads to infant mortality and lowers life expectancy [9,10]. At the same time, exposure to air pollution and its negative health implications impose additional socio-economic costs, such as reductions in hours worked [11,12], productivity [13], and even absenteeism from school [14]. These socio-economic costs can further lead to bad health due to the impact that socio-economic conditions have on health [15], which makes this an especially difficult problem to tackle, especially in developing countries and in communities characterized by low socio-economic conditions.

However, most of this literature relies on short-term cross-sectional data, which, on one hand, does not allow causal inference, and on the other hand, describe short-term effects of pollution, which completely ignores the possibility that some of the health effects may not even develop in the short-run or may be persistent over long periods of time, even after the air quality improves.

First, most of the medical literature that establishes links between air pollution and human health relies on observational data, since randomized controlled trials cannot be ethically performed to establish true causal effects of pollution exposure. Therefore, the results of this literature are merely correlational in nature and possibly biased by confounding factors. However, a more recent strand of literature in social sciences does attempt to correct this issue by employing more advanced methods of causal inference that rely on exogenous variation in pollution due to certain policy changes or natural factors that act as a randomized treatment and can provide causal estimates. While this literature is still thin and under development, there are several studies that were successful at using such causal inference methods to quantify the negative effects of pollution on respiratory health [16], mental health [17], and even infant mortality [18].

Second, most of the documented effects represent immediate or short-term effects of pollution. There is only a limited number of studies documenting the true long-term (persistent) effects of air pollution. Of note is the fact that even studies that claim to study long-term effects, do not actually document such effects but merely the presence of short-term effects over long periods of time, which can be more accurately described as short-term effects of long-term exposure to pollution. For instance, there are studies documenting reductions in lung function [19], increases in arterial blood pressure [20], and reductions in hemoglobin levels [21] due to long-term pollution exposure. However, none of these results document the persistence of such effects over time, after the pollution exposure ends.

The objective of our study is to address both shortcomings that we mentioned above by documenting a causal link between exposure to severe but transitory episodes of air pollution and human health. We are specifically interested in effects that are persistent over long periods of time—years and decades after the initial pollution exposure. This is extremely important for policymakers because pollution exposure may have consequences that last even after the air quality improves and so mitigation efforts should not stop with the improvement of air quality. Our paper extends previous results [16] that showed reductions in lung capacity and general health status, as measured 10 years after exposure to pollution. We significantly extend the time horizon and show that some effects persist even longer, although there does seem to be some recovery over time. We find that exposure to air pollution reduces the lung capacity of respondents and increases the likelihood of poor general health. These effects can still be observed almost 2 decades after the exposure to pollution, but there is some recovery over time. We also find basic socioeconomic factors, such as education and per capita expenditures, to be good mitigators.

## 2. Methods and Data

Most of the literature documenting the negative health consequences of air pollution are comprised of correlational studies that are not able to capture true causality implications. These correlations are still useful in painting a general picture, but not extremely accurate due to the presence of many potential confounders. A small number of studies do try to address this issue by making use of natural experiments or certain policy changes that introduce the equivalent of a random treatment in a certain population. These types of exogenous shocks are ideal tools for scientists to pinpoint causal effects.

In the summer of 1997, massive forest fires covered large parts of Indonesia and ran wild for months. An extremely dry and windy season that year led to the rapid spread of these fires and the eventual loss of control until the start of the rainy season in November. Large parts of the country were covered in smoke between August and November and even neighboring countries were affected by this massive episode of air pollution [22]. This pollution shock is an ideal source of exogenous variation and was already used as an identification strategy to document the causal effects of air pollution on human health in previous studies [12,16,17,18].

We use satellite data on pollution exposure. The data was generated by the Total Ozone Mapping Spectometer (TOMS) and was interpolated using GPS coordinates for each community. We closely followed the methodology used in the existing literature [12,16] to assemble our pollution exposure variable. The TOMS data represents an aerosol index that is calculated from the amount of light that different pollutants absorb or reflect. This type of data has been found to closely track data generated by traditional ground-based pollution monitors [23]. The scale of the index is between the values of −2 and 7. Negative numbers represent particles that do not absorb light such as sulfates. Positive numbers represent light-absorbing particles such as smoke. During the 1997 fires, the observed values of the index are all in the positive domain, with large jumps compared to the previous years. For instance, previous studies [12] report the mean value of the pollution index in 1996 was 0.089, whereas in 1997 it was 0.691, which is an almost 8-fold increase. The maximum value of the index in 1996 was 0.395, while in 1997 the maximum value was 4.841, which represents a 12-fold increase. While this data is unitless and does not allow for meaningful quantitative interpretations and comparisons, it has the big advantage of covering large territories and long periods of time and therefore can still be used for qualitative studies.

We link this pollution exposure data with health and socio-economic data from the Indonesia Family Life Survey (IFLS). IFLS is a large longitudinal survey that is representative of roughly 83% of the Indonesian population. Only about 17% of the population living in the outer provinces of Indonesia was not surveyed by IFLS, due to cost considerations. The first wave of the IFLS survey was fielded in 1993, with subsequent waves in 1997, 2000, 2007, and 2015. Attrition rates between survey waves are very low in IFLS, especially given its time coverage of over 2 decades. This makes IFLS data extremely valuable for our purpose since we can track thousands of respondents over long periods of time.

Our paper extends the previous work of Kim et al. [16] by adding the last wave of IFLS data, and thus being able to capture some interesting patterns of recovery from pollution. This previous work uses similar data and finds significant effects of pollution on human health that persist even ten years after the initial exposure. By adding the newly available data from the most recent IFLS wave, we are able to almost double the period of time during which we measure health outcomes and observe their dynamics. The last IFLS wave was fielded between August 2014 and April 2015. Some interesting patterns emerge from this analysis.

We estimate a basic health equation of the form:

$$H_{ij}^{t}=\alpha H_{ij}^{1997}+\beta {Pollution}_{j}^{1997}+\gamma X_{i}+\epsilon_{ij}$$

where $H_{ij}^{t}$ denotes the health of respondent *i* living in community *j* at period *t*, $H_{ij}^{1997}$ represents the baseline health of the respondent before exposure to pollution, ${Pollution}_{j}^{1997}$ represents the pollution index for community *j* during the 1997 fires, *X* is a vector of socioeconomic control variables, and *ε_ij_* is the error term representing the unobservables uncorrelated with the regressors. We use two main health outcomes: lung capacity and the general health status of respondents. We also analyze the health response at two different times: in 2007 and 2014. We use the Ordinary Least Squares (OLS) method with robust standard errors to estimate our parameters of interest.

## 3. Results

We focus on lung capacity as our most important health measure that can be affected by air pollution. The fine particulate matter contained in smoke is inhaled into the lungs, which leads to a number of respiratory issues [24]. Measuring lung capacity is the gold standard test to diagnose restrictive lung disease. Lung capacity and low inspiratory capacity have been shown to be major risk factors for COPD [25], and a good predictor for hyperinflation and all cause-mortality [26,27]. Lung capacity is also an objective measure of health that leaves no room for subjectivity or other types of behavioral and psychological effects. Trained nurses, using the Personal Best Peak Flow Meter, collected lung capacity in the IFLS. Three separate measurements were taken and we used the average of these three measurements. The results of the regression analysis are presented in Table 1:

Table 1 contains 2 different sets of results. The first estimates in the table, which use the 2007 health data, mainly replicate previously documented results and point to the persistent long-term effects of air pollution on lung capacity, as measured in 2007 (ten years after the exposure to the 1997 pollution episode). There are some minor differences in magnitudes between our replication and the original study by Kim et al. [16], which are due to slight differences in sample sizes and composition between the two studies. Overall, however, we are able to replicate their original results fairly accurately. For instance, the main coefficient of interest, which is the effect of pollution exposure on lung capacity, has an estimated magnitude of −5.370 in the original study and −5.997 in our replication.

The second set of results, which represents the main contribution of this paper, extends the original study and also the overall existent literature by estimating the persistent effects of air pollution on lung capacity, as measured in 2014 (17 years after exposure). To our knowledge, there is no other study in the literature that estimates the causal effects of air pollution on lung capacity and the persistence of these negative consequences over such an extended time period.

There are two important results that emerge from this analysis: first, the negative effects of pollution exposure are extremely long-lived, and second, there does seem to be some recovery over time. As seen in the table, the main coefficient of interest is statistically significant even 17 years after the pollution exposure. However, the estimated magnitude of the effect in 2014 is roughly half the size of the estimated magnitude of the effect in 2007, which points to this pattern of recovery. Exposure to an additional unit of pollution in 1997 leads to a loss of roughly six units of lung capacity in 2007 (10 years after), but only to a loss of roughly three units of lung capacity in 2014 (17 years after). Due to the fact that our measure of pollution is an index and not an actual amount or concentration of PM 2.5 particles, it is hard to meaningfully interpret the magnitudes of these effects in a quantitative sense. However, qualitatively and relatively speaking, since both effects are measured using the same scale and the same sample of respondents, it is clear that the effects diminish over time. At the same time, the coefficient for baseline lung capacity (as measured prior to the pollution exposure) increases in magnitude over time. This is, again, suggestive of the fact that, although the negative effects of air pollution are extremely long-lived, they do somewhat diminish with the passage of time and respondents’ current lung capacity gets to be more in line with their baseline lung capacity as time passes and the effects of the pollution exposure diminish.

As an additional health measure, we also consider the general health status (GHS), which is a subjective measure of health that is collected by the IFLS. GHS is a self-reported health measure that, although prone to subjective biases, can still be a very good predictor of objective health. Especially in longitudinal studies, where respondents self-report their GHS over time and we can condition on baseline GHS, these subjective biases are somewhat controlled for. The advantage of measures such as GHS is that they can proxy for a large variety of health issues and they have been found to predict quite well future mortality [28,29]. IFLS respondents were asked to score their general health as either very healthy, somewhat healthy, somewhat unhealthy, or unhealthy. We grouped these responses into two main categories and coded an indicator variable to take a value of 1 for those respondents who rated their general health status as unhealthy or somewhat unhealthy. Therefore, this represents respondents in poor general health and the estimated coefficient will represent marginal increases in the probability of being in poor general health status. These estimations are presented in Table 2.

The effects estimated in the table present a similar picture to the estimated effects on lung capacity. Exposure to the severe air pollution episode in 1997 has effects that are extremely long-lived, but somewhat diminish over time. Exposure to an additional unit of pollution in 1997 leads to an increase of about 4.3% in the probability of being in poor general health in 2007 (10 years after exposure) and to an increase of only about 3% in the probability of being in poor general health in 2014 (17 years after exposure). At the same time, the estimated effect of the baseline GHS increases over time, which again is indicative of the same pattern of recovery: current health status depends more on baseline health status and less on the exposure to pollution, as time passes. However, just like in the case of lung capacity, this recovery seems to be very slow and the effects of pollution exposure are still significant even after almost 2 decades. As before, the estimated effects after 10 years are mainly a replication of previous work done by Kim et al. [16], and we do confirm their original results. Although the sample sizes and composition are slightly different, our main estimated coefficient is 4.27%, which is remarkably close to their original estimate of 4.56%. Our main contribution remains the extension of the time horizon under analysis and documentation of the recovery pattern.

## 4. Discussion

A small number of recent studies [16,17,18] have started to investigate the persistence of negative health consequences resulting from air pollution. Due to data limitation, such long-term studies are rare. Not only do such studies need respondents to be tracked over time for years and even decades, which is costly and presents many logistical difficulties, but also the presence of confounding factors increases with the passage of time, which can bias the results. However, carefully designed studies that use longitudinal data and make use of causal inference methods to limit the potential bias of the estimates, present a particularly important issue related to the relationship between air quality and human health. These studies show that the effects of air pollution do not disappear once the air quality is improved, but can linger for years.

We are able to replicate and confirm the results from a previous study [16] that shows the negative health effects of exposure to air pollution linger even 10 years after the initial exposure. Our study then extends this thin literature considerably by using a longer longitudinal survey that spans almost 2 decades after a major pollution episode that occurred in Indonesia and that exposed the local population to severe amounts of particulate matter. We show that these negative health consequences last even longer than initially documented. We find that pollution exposure reduced lung capacity and increased the likelihood of being in poor general health, and these effects persist even 17 years after exposure to pollution. Although there is some recovery over time, the effects are still highly significant. Our results, therefore, present an important contribution to scientific knowledge by almost doubling the time horizon over which these effects were previously documented.

At the same time, the pollution exposure in Indonesia was primarily determined by natural forces (such as winds and other climate-related forces), which are unlikely to be correlated with certain socioeconomic aspects that may also affect health. This natural experiment allows us to pinpoint true causal estimates, not merely correlational ones. This is one other important differentiation factor between our study and the vast majority of studies in medical fields that document associations between air pollution and different measures of human health. If the pollution exposure is not random, but is in fact correlated to certain demographic or socio-economic characteristics, the estimated results can be biased due to these confounding factors. For instance, poorer households, who are in poorer health to begin with, also live in areas that are more likely to be heavily polluted, which can lead to overestimation bias. However, natural experiments, such as the one we are using, are unlikely to lead to such correlations and estimate bias since one cannot argue that the winds push the smoke clouds in a purposeful way over specific socio-economic groups and avoid others. Unfortunately, while the medical literature is saturated with correlational studies that can only show associations between human health and air quality, the social science literature trying to establish true causal inference is not that well developed. This is primarily because natural experiments do not occur that often on a scale large enough that can be then matched with survey data containing health information. The Indonesian episode we are using has been vastly used as an identification mechanism in a variety of studies in the social science literature.

Lastly, while the effects we document are important, and their persistence over such long periods of time is striking, the data we have is not sufficient to predict how long will such effects persist. In other words, it is not yet clear whether there will ever be a full recovery for those exposed to pollution. While we do observe some recovery between the 10th year mark and the 17th year mark, our data does not yet show a complete disappearance of these effects. Future research needs to be carried out in the future, when new data becomes available, to document the time horizon over which these health effects persist. Properly understanding these dynamics of health over time is extremely important for policymakers, especially for local authorities who have control over the agricultural practices that sparked this pollution in the first place. Especially in developing countries, slash-and-burn techniques are still an extremely common way to clear agricultural land cheaply. Moreover, these policymakers and local authorities may wrongly assume that burning vegetation and polluting the air is only a temporary problem. Our results show this may not be the case and if the pollution is serious enough, serious health effects can last much longer than previously believed and affect the local population in a variety of ways, since bad health has a series of negative consequences on many other socio-economic aspects of human life.

## 5. Conclusions

This paper documents extremely long-lived negative consequences of exposure to air pollution. Unlike the literature that documents the effects of long-term exposure to pollution, this paper documents the effects of temporary exposure to a one-time severe pollution episode. These effects turn out to be extremely persistent. Although there does seem to be a pattern of recovery, some effects still linger almost two decades after the initial exposure.

Our paper adds to the well-established literature and raises serious public health concerns regarding the persistence of these negative health consequences. Of particular concern is the fact that slash-and-burn practices, such as the ones that sparked the Indonesian fires in 1997, remain extremely common, especially in rural communities in developing nations. It is also the same rural communities that are usually resource-constrained, lacking access to medical care, and severely exposed to indoor air pollution from burning vegetation as fuels, which further puts a strain on their health. Policymakers should, therefore, address these health inequalities by trying to minimize exposure to all various sources of air pollution, without assuming that transitory episodes of pollution do not matter. This paper strengthens the evidence that even a passing episode of pollution, which only lasted a few months, can have devastating consequences to health that can last decades.

At the same time, however, there does seem to be some recovery over time, which is encouraging. Furthermore, socioeconomic factors, such as education and per capita expenditures, seem to be extremely good mitigators. Socioeconomic factors play a theoretical double role: they allow people to avoid exposure to pollution during the initial shock and they also allow exposed individuals to access better medical care, which can help the recovery process. Therefore, policymakers should also continue to fight against poverty and socioeconomic disparities, which can exacerbate the public health problems created by air pollution.

## Figures and Tables

**Table 1:** Lung Capacity Regression Results.

Explanatory Variables	Dep. Variable: Lung Cap. In 2007	Dep. Variable: Lung Cap. In 2014
Pollution 1997	−5.997327 *** (1.27721)	−3.12409 ** (1.363444)
Age	−5.431433 *** (0.3311479)	−8.288143 *** (0.4148818)
Age Squared	0.0342484 *** (0.0036178)	0.045701 *** (0.003938)
Education	2.120115 *** (0.2194304)	2.162752 *** (0.2194361)
Log PCE	3.068381 ** (1.311647)	6.516261 *** (1.294435)
Outside Kitchen	3.534371 ** (1.673195)	2.564004 (1.799692)
Outside Water	−3.185497 (1.995559)	−1.603927 (2.137372)
Lung Capacity in 1997	0.581728 *** (0.0090033)	0.627679 *** (0.0097219)
Constant	252.8612 *** (19.12685)	352.2007 *** (17.58381)
Sample Size	10,483	10,483
F-stat	833.53 (*p* = 0.0000)	1081.84 (*p* = 0.0000)
R-square	0.3877	0.4405
Robust standard errors in parentheses. **—significant at 5% level; ***—significant at 1% level		
The regression results using the 2007 lung capacity data represents a replication of Kim et al. (2017) [16], while the results using the 2014 lung capacity data represents the extension and contribution of the current study.		

**Table 2:** General Health Status (GHS) Regression Results.

Explanatory Variables	Dep. Variable: Poor GHS. In 2007	Dep. Variable: Poor GHS. In 2014
Pollution 1997	0.0426577 *** (0.0050672)	0.0303704 *** (0.0055657)
Age	−0.0000745 (0.0007535)	−0.0008773 (0.0010255)
Age Squared	0.000033 *** (0.0000101)	0.0000588 *** (0.0000112)
Education	−0.0022316 (0.0007537)	−0.0054677 *** (0.0008656)
Log PCE	0.0075451 * (0.0043726)	−0.001981 (0.0050125)
Outside Kitchen	0.0004769 (0.0068785)	−0.0210083 *** (0.0070284)
Outside Water	−0.0188559 *** (0.0068785)	−0.0085405 (0.0082364)
Poor GHS in 1997	0.0925645 *** (0.0121173)	0.1194477 *** (0.0136485)
Constant	−0.0192876 (0.0561307)	0.2132178 *** (0.0703729)
Sample Size	15,022	15,022
F-stat	49.52 (*p* = 0.0000)	112.87 (*p* = 0.0000)
R-square	0.0350	0.0624
Robust standard errors in parentheses. *—significant at 10% level; ***—significant at 1% level		
The regression results using the 2007 GHS data represents a replication of Kim et al. (2017) [16], while the results using the 2014 GHS data represents the extension and contribution of the current study.		

## Data Availability

The IFLS data analyzed in the study is publicly available at https://www.rand.org/well-being/social-and-behavioral-policy/data/FLS/IFLS.html.
